# Area of the pressure-strain loop during ejection as non-invasive index of left ventricular performance: a population study

**DOI:** 10.1186/s12947-019-0166-y

**Published:** 2019-08-05

**Authors:** Nicholas Cauwenberghs, Mahdi Tabassian, Lutgarde Thijs, Wen-Yi Yang, Fang-Fei Wei, Piet Claus, Jan D’hooge, Jan A. Staessen, Tatiana Kuznetsova

**Affiliations:** 1 Research Unit Hypertension and Cardiovascular Epidemiology KU Leuven Department of Cardiovascular Sciences, University of Leuven, Leuven, Belgium; 2 Division of Cardiovascular Imaging and Dynamics, Department of Cardiovascular Sciences, University of Leuven, Leuven, Belgium

**Keywords:** Echocardiography, Hypertension, Ventricular-arterial coupling, Longitudinal strain, Ejection work density

## Abstract

**Background:**

Previous studies highlighted the usefulness of integrating left ventricular (LV) deformation (strain) and hemodynamic parameters to quantify LV performance. In a population sample, we investigated the anthropometric and clinical determinants of a novel non-invasive index of LV systolic performance derived from simultaneous registration of LV strain and brachial pressure waveforms.

**Methods:**

Three hundred fifty-six randomly recruited subjects (44.7% women; mean age, 53.9 years; 47.5% hypertensive) underwent echocardiographic and arterial data acquisition. We constructed pressure-strain loops from simultaneously recorded two-dimensional LV strain curves and brachial pressure waveforms obtained by finger applanation tonometry. We defined the area of this pressure-strain loop during ejection as LV ejection work density (EWD). We reported effect sizes as EWD changes associated with a 1-SD increase in covariables.

**Results:**

In multivariable-adjusted analyses, higher EWD was associated with age, female sex and presence of hypertension (*P* ≤ 0.0084). In both men and women, EWD increased independently with augmentation pressure (effect size: + 59.1 Pa), central pulse pressure (+ 65.7 Pa) and pulse wave velocity (+ 44.8 Pa; *P* ≤ 0.0006). In men, EWD decreased with relative wall thickness (− 29.9 Pa) and increased with LV ejection fraction (+ 23.9 Pa; *P* ≤ 0.040). In women, EWD increased with left atrial (+ 76.2 Pa) and LV end-diastolic (+ 43.8 Pa) volume indexes and with E/e’ ratio (+ 51.1 Pa; *P* ≤ 0.026).

**Conclusion:**

Older age, female sex and hypertension were associated with higher EWD. Integration of the LV pressure-strain loop during ejection might be a useful tool to non-invasively evaluate sex-specific and interdependent effects of preload and afterload on LV myocardial performance.

**Electronic supplementary material:**

The online version of this article (10.1186/s12947-019-0166-y) contains supplementary material, which is available to authorized users.

## Background

The performance of the left ventricle (LV) is determined by its intrinsic contractility (inotropy), by the tension on the LV wall at end-diastole (preload) and by the load against which the LV needs to eject blood (afterload) [1]. If myocardial and vascular properties are matched, the heart can adequately respond to changes in pre- and afterload and regulate cardiac output and blood pressure (BP). However, the adaptive response to increased afterload appears impaired in patients with chronic hypertension [2] and with symptomatic heart failure [2, 3].

In line, community-based studies demonstrated the detrimental impact of increased afterload on LV structure and function [4–7]. Indeed, a long-term increased afterload and, consequently, a chronically increased cardiac performance lead to adverse LV maladaptation and increased LV oxygen requirements [8]. In particular, older women appear susceptible to the detrimental effects of increased pulsatile load on LV diastolic function [4, 6, 7], which might be explained by the higher aortic stiffness, enhanced LV systolic performance, higher preload sensitivity and lower LV compliance in women as in men [9].

Echocardiographic techniques such as two-dimensional (2D) speckle tracking allow quantification of the relative myocardial deformation (i.e. strain) [10]. Furthermore, LV strain and arterial function can be assessed simultaneously in a non-invasive way [11]. In fact, recent developments in construction and quantification of pressure-strain loops illustrates the pressing need for a non-invasive, clinical tool that integrates both LV deformation and its loading conditions [2, 12]. Several indexes derived from these simultaneous recordings were suggested to reflect the interaction between the heart and the arteries (i.e. ventricular-arterial coupling, VAC) [2, 12]. For instance, we previously constructed LV pressure-strain loops from simultaneously recorded LV strain curves and carotid pressure waveforms, and defined the area of these loops during ejection as the LV ejection work density (EWD) [2].

In fact, EWD might better reflect LV systolic performance than peak LV deformation, as it integrates the instantaneous deformation as well as the instantaneous afterload against which the myocardium has to shorten [2, 11]. However, we should better understand this LV performance index before evaluating its utility in clinical decision-making. Therefore, we investigated in a population sample the anthropometric and clinical determinants of EWD. We also explored the relationship of EWD with indexes reflecting LV structure, LV diastolic function and arterial stiffness.

## Materials and methods

### Study participants

The Flemish Study on Environment, Genes and Health Outcomes (FLEMENGHO) was approved by the Ethics Committee of the University of Leuven. We randomly recruited a family-based population sample in northern Belgium as described before [5]. All subjects provided written informed consent. Between 2011 and 2016, we performed a VAC protocol including simultaneous echocardiography and finger applanation tonometry in 405 participants. We excluded 49 subjects from statistical analysis because of a history of myocardial infarction or ischemic heart disease (*n* = 14), atrial fibrillation (*n* = 4) or symptomatic heart failure (n = 1), or because of insufficient quality of the echocardiograms (*n* = 6) or finger pressure waves (*n* = 24). In total, we thus analysed data from 356 participants.

### Echocardiography

Echocardiography and arterial phenotyping were performed after the subject had rested for at least 15 min in supine position. Details on the echocardiography, arterial phenotyping and other measurements are provided as *Data Supplement*.

*Data acquisition -* Briefly, one experienced physician (T.K.) did the ultrasound examination using a Vivid E9 (GE Vingmed, Norway) interfaced with a 2.5- to 3.5-MHz phased-array probe, in accordance to recommendations [13] and previous reports [5].

During echocardiography, we recorded continuous finger pressure waves at the subject’s right middle-finger using a Finometer Pro (Finapres Medical Systems, The Netherlands), which were converted to brachial pressure curves by a validated transfer function implemented in the Finometer software [14].

*Off-line analysis -* One observer (TK) analyzed the echocardiograms using EchoPac software (GE Vingmed). End-diastolic LV dimensions were used to calculate relative wall thickness (RWT) and LV mass. We measured transmitral peak early (E) and late (A) diastolic velocities as well as early (e’) and late (a’) diastolic peak mitral annular velocities at 4 acquisition sites (septal, lateral, inferior and posterior). The E/e’ ratio, a surrogate of LV filling pressure, was transmitral E peak divided by e’ averaged from the 4 acquisition sites. Two observers (N.C and T.K.) measured LV global longitudinal strain (LS) using myocardial speckle-tracking software (Q-analysis, GE Vingmed) as described before [15]. We used peak systolic, mid-wall global LS for statistical analysis. Additional file 1: Figure S1 shows the relative inter-observer variability of individual segmental LS values.

**Fig. 1:**
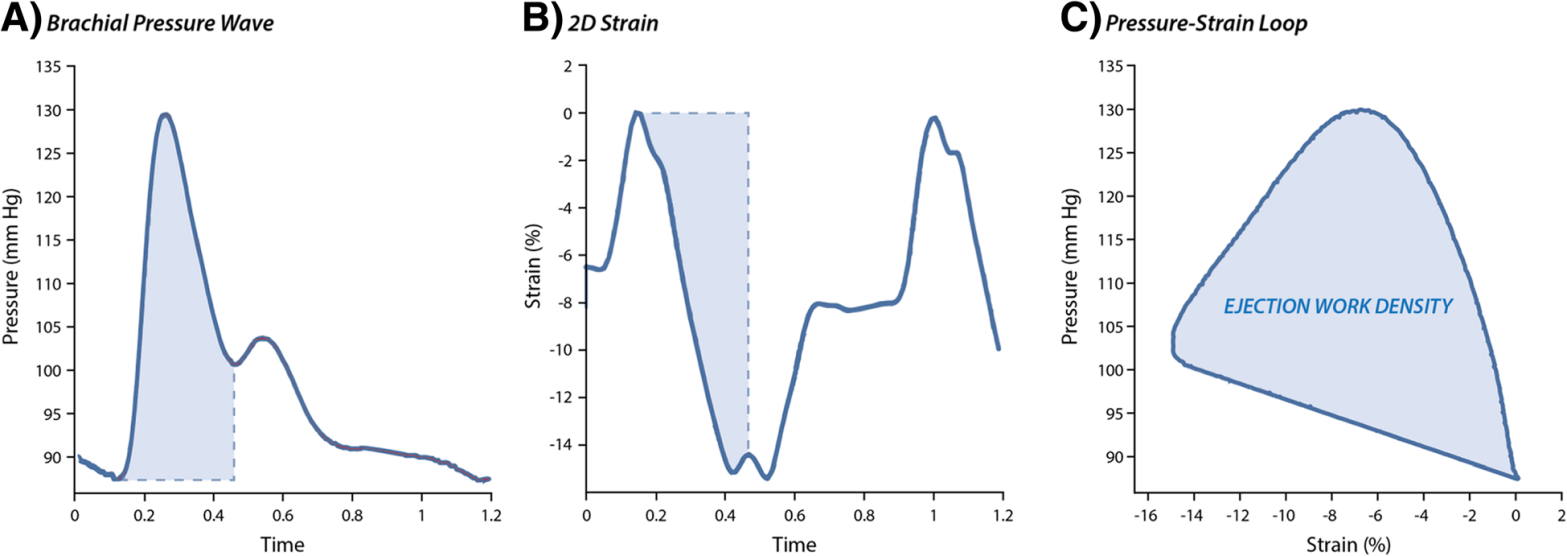
Non-Invasive Assessment of the LV Ejection Work Density**.** Using echocardiography and simultaneous applanation tonometry, we derived brachial artery pressure wave (panel **a**) and two-dimensional LV strain curves (panel **b**) to construct the pressure-strain loop (panel **c**). The myocardial work index was calculated as the area of the pressure-strain loop during LV ejection (filled area in panel **c**). LV indicates left ventricular

*VAC analysis –* We post-processed the simultaneously recorded LV deformation curves and pressure waves using a custom Matlab algorithm (The MathWorks, Inc., USA). The performance of the software was validated in 50 subjects using intermediate and final quality checkpoints and is available upon request from the corresponding author (Additional file 1: Figure S2). First, we constructed pressure-strain loops by plotting the calibrated brachial pressure wave against the global LS curve (Fig. 1). Next, we calculated EWD as the area of the pressure-strain loop during LV ejection, representing the cumulative work density on the muscle that instantaneously shortens a given amount (i.e. change in strain) against an instantaneous pressure (Fig. 1) [2]. We averaged EWD from apical 4 and 2 chamber views (Additional file 1: Figure S3). We additionally included the average EWD standardized by RWT in sensitivity analyses to better account for LV wall stress.

### Arterial measurements

We recorded carotid, femoral and radial arterial waveforms using a SPC-301 micromanometer (Millar Instruments Inc., USA) linked to a computer running SphygmoCor software (AtCor Medical Pty. Ltd., Australia). Pulse waves were calibrated by the supine brachial BP measured immediately before tonometry. From radial signals, SphygmoCor software constructed the aortic (central) pulse wave using a validated generalized transfer function. Central pulse pressure (PP) was central systolic minus diastolic pressure. Augmentation pressure (AP) was the pressure difference between the first and second shoulder of the central waveform. In 157 men and 117 women, we measured aortic pulse wave velocity (PWV), the non-invasive gold standard of arterial stiffness, as the carotid-femoral distance divided by the carotid-femoral pressure transit time [16].

### Other measurements

Conventional BP was the average of 5 auscultatory readings obtained with the subject in seated position. Hypertension was defined as a BP of at least 140 mmHg systolic or 90 mmHg diastolic or the use of antihypertensive drugs. Participants on antihypertensive therapy were defined as having either uncontrolled (BP > 140/90) or controlled (BP ≤ 140/90) hypertension. Diabetes mellitus was determined by self-report, a fasting glucose level of at least 126 mg/dL, or the use of antidiabetic agents.

### Statistical analysis

We used SAS software v9.4 (SAS Institute, Cary, NC) for database management and statistical analysis. We compared means and proportions using a large sample z-test and χ2-test, respectively. Statistical significance was a two-sided *P* value < 0.05. Using mixed models, we assessed multivariable-adjusted associations of EWD with anthropometric and clinical characteristics, hemodynamics, arterial stiffness and echocardiographic indexes of LA volume index (LAVi) and LV structure and function. All models were adjusted for age, sex, heart rate and body height and weight, and accounted for family clusters modelled as a random effect. We reported multivariable-adjusted regression coefficients for EWD per 1-SD increase in the covariable. We repeated regression analyses for men and women separately.

## Results

### Characteristics of participants

The 356 participants (44.7% women) included 169 (47.5%) hypertensive subjects, of whom 92 (54.4%) were on antihypertensive drug treatment. Mean age was 53.9 ± 13.7 years. Tables 1 and 2 list the clinical, arterial and echocardiographic characteristics of the study cohort by sex. EWD averaged 635.8 ± 179.6 Pa in men and 782.3 ± 224.7 Pa in women (*P* < 0.0001; Table 2).

**Table 1:** Clinical characteristics of 356 participants by sex

Characteristic	Men (*n* = 197)	Women (*n* = 159)	*P* value
*Anthropometrics*			
Age, y	53.2 ± 14.6	54.8 ± 12.5	0.27
Body mass index, kg/m^2^	27.3 ± 3.56	26.5 ± 4.32	0.060
Systolic BP, mm Hg	131.6 ± 14.8	129.3 ± 17.3	0.18
Diastolic BP, mm Hg	85.3 ± 9.33	81.4 ± 8.85	< 0.0001
Pulse pressure, mm Hg	46.3 ± 12.8	48.0 ± 13.8	0.23
Mean arterial pressure, mm Hg	100.7 ± 9.72	97.4 ± 10.5	0.0020
Heart rate, bpm	58.5 ± 8.98	60.6 ± 8.71	0.026
*Questionnaire data*			
Current smoking, n (%)	31 (15.7)	31 (19.5)	0.35
Drinking alcohol, n (%)	119 (60.4)	37 (23.3)	< 0.0001
Hypertensive, n (%)	100 (50.8)	69 (43.4)	0.17
Treated for hypertension, n (%)	54 (27.4)	38 (23.9)	0.45
β-blockers, n (%)	25 (12.7)	18 (11.3)	0.69
ACE or ARB, n (%)	26 (13.2)	10 (6.3)	0.032
CCB, n (%)	18 (9.1)	7 (4.4)	0.082
Diuretics, n (%)	18 (9.1)	16 (10.1)	0.77
History of diabetes, n (%)	8 (4.1)	8 (5.0)	0.66
*Biochemical data*			
Serum creatinine, μmol/L	81.6 ± 13.0	66.2 ± 17.9	< 0.0001
Total cholesterol, mmol/L	4.84 ± 0.93	5.28 ± 0.96	< 0.0001
Serum insulin, μmol/L	5.20 (2.20–12.0)	4.67 (2.00–10.7)	0.10

Values are mean (±SD), number of subjects (%) or median (10–90% percentile interval). *ACE* indicates angiotensin-converting enzyme, *ARB* angiotensin-receptor blockers, *bpm* beats per minutes, *CCB* calcium channel blockers

**Table 2:** Arterial and echocardiographic characteristics of 356 participants by sex

Characteristic	Men (*n* = 197)	Women (*n* = 159)	*P* value
*Arterial characteristics*			
AP, mm Hg	10.3 ± 8.00	15.3 ± 9.55	< 0.0001
Central PP, mm Hg	40.3 ± 12.1	44.5 ± 14.3	0.0059
PWV, m/s^a^	8.18 ± 1.90	7.94 ± 1.94	0.30
*LV end-diastolic dimensions*			
Internal diameter, cm	5.27 ± 0.40	4.85 ± 0.35	< 0.0001
Septal wall thickness, cm	1.05 ± 0.13	0.92 ± 0.12	< 0.0001
Posterior wall thickness, cm	0.99 ± 0.11	0.88 ± 0.094	< 0.0001
Relative wall thickness	0.39 ± 0.053	0.37 ± 0.043	0.0041
LV mass index, g/m^2^	101.9 ± 20.8	86.2 ± 15.4	< 0.0001
*Echocardiographic volumes*			
LA volume index, ml/m^2^	28.0 ± 8.46	26.1 ± 6.70	0.021
LV EDV index, ml/m^2^	55.9 ± 9.42	46.6. ± 8.12	< 0.0001
LV ESV index, ml/m^2^	23.0 ± 4.57	18.3 ± 3.83	< 0.0001
LV Stroke volume, ml	66.4 ± 13.6	50.0 ± 11.7	< 0.0001
LV Ejection fraction, %	58.9 ± 4.99	60.7 ± 5.11	0.0010
*LV diastolic function*			
E peak, cm/s	63.1 ± 13.9	69.3 ± 14.9	< 0.0001
A peak, cm/s	55.3 ± 14.1	63.0 ± 15.4	< 0.0001
E/A ratio	1.22 ± 0.44	1.18 ± 0.44	0.33
e’ peak, cm/s	9.97 ± 3.29	10.0 ± 3.15	0.94
a’ peak, cm/s	9.76 ± 2.09	9.31 ± 1.88	0.036
E/e’ ratio	6.76 ± 1.87	7.42 ± 2.32	0.0038
*LV strain*			
Global LS	18.5 ± 1.84	20.3 ± 1.87	< 0.0001
*Ejection work density*			
EWD, Pa	635.8 ± 179.6	782.3 ± 224.7	< 0.0001
EWD / RWT, Pa	1665.2 ± 498.4	2109.1 ± 592.1	< 0.0001

Values are mean (±SD). ^a^Data on arterial stiffness was available in 157 men and 117 women. AP indicates augmentation pressure, *EDV* end-diastolic volume, *ESV* end-systolic volume, *EWD* ejection work density, *LA* left atrial, *LS* longitudinal strain, *LV* left ventricular, *PP* pulse pressure, *PWV* pulse wave velocity

### Anthropometric and clinical determinants of EWD

**Fig. 2:**
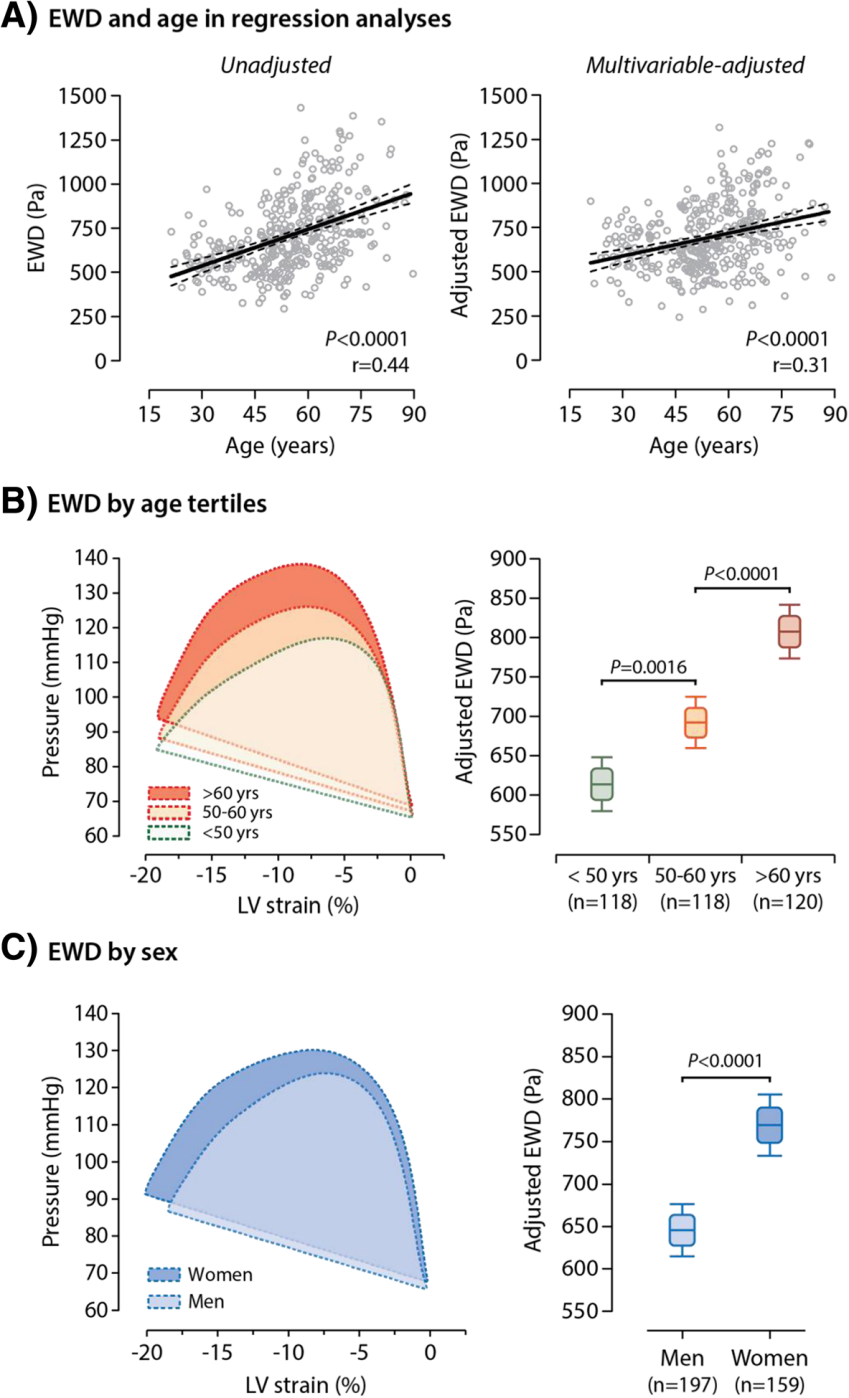
The Ejection Work Density (EWD) in Relation to Age (Panels **a** and **b**) and Sex (Panel **c**). Full and dotted lines in Panel **a** represent the linear regression line and 95% confidence band, respectively. Boxplots present the adjusted mean EWD and 5–95% and 25–75% confidence limits by age tertiles (Panel **b**) and sex (Panel **c**). Adjustments in EWD accounted for the variance explained by age (except in **a**-**b**), sex (except in **c**), hypertension, heart rate and body height and weight

EWD increased significantly with age in both unadjusted and multivariable-adjusted analyses (*P* < 0.0001; Fig. 2a). As shown in Fig. 2b, the pressure-strain loop extended greater along the pressure axis with only little fluctuation in peak global LS in older subjects as compared to younger ones. We observed similar age-related changes in pressure-strain loop and EWD in both men and women (Additional file 1: Figure S4*).* Of note, EWD was higher in postmenopausal than in premenopausal women (*P* < 0.0001; Additional file 1: Figure S5).

**Fig. 3:**
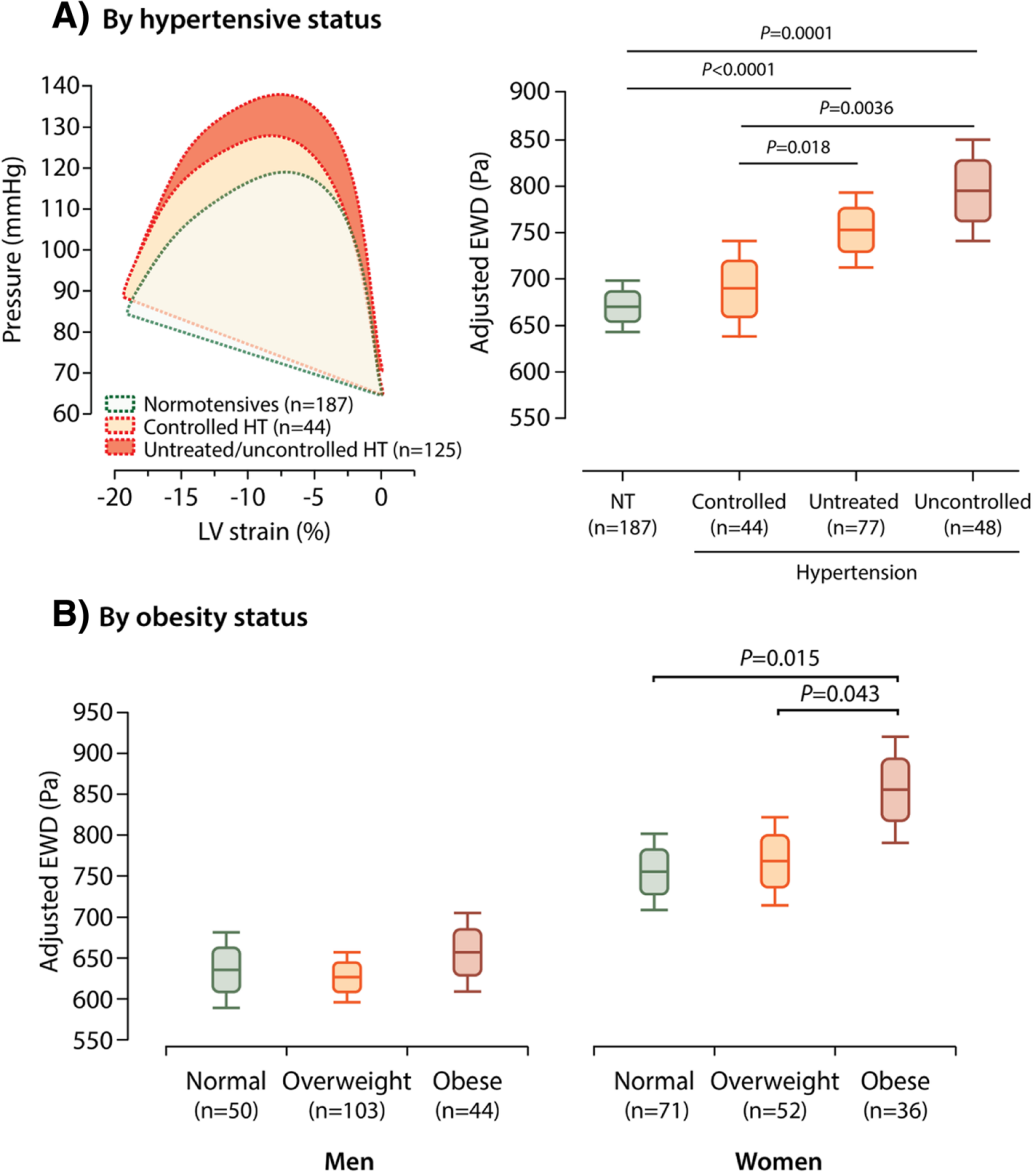
The Ejection Work Density (EWD) in Relation to Hypertension (Panel **a**) and Obesity (Panel **b**). Boxplots present the adjusted mean EWD and 5–95% and 25–75% confidence limits. Adjustments in EWD accounted for the variance explained by age, sex (except in B) and heart rate. HT indicates hypertension; LV, left ventricular; NT, normotensive

On average, after adjustment for age, heart rate and body size, the pressure-strain loop area during ejection was in its entirety broader in women than in men (Fig. 2c). Hence, averaged EWD was significantly higher in women as compared to men (*P* < 0.0001; Fig. 2c). Furthermore, adjusted EWD was significantly greater in participants whose hypertension was either untreated (*P* = 0.018) or uncontrolled (*P* = 0.0036) as compared to subjects with controlled hypertension (Fig. 3a). Of note, EWD did not differ between normotensives and subjects with controlled hypertension (*P* = 0.49; Fig. 3a).

After full adjustment, EWD decreased with the use of β-blockers (− 65.4 Pa; *P* = 0.046), but was not related to the use of ACE-inhibitors/ARBs, calcium channel blockers or diuretics (*P* ≥ 0.51). In addition, after accounting for sex and age, EWD was significantly higher in obese than in non-obese women (*P* ≤ 0.043; Fig. 3b). In both unadjusted and fully-adjusted analysis, EWD was not associated with heart rate (*P* ≥ 0.23) or with smoking (*P* ≥ 0.22).

Our findings were consistent for the 4 and 2 chamber EWDs analyzed separately (data not shown).

### Associations of EWD with arterial hemodynamics and stiffness

Table 3 presents the overall and sex-specific multivariable-adjusted estimates (95% CI) for EWD associated with a 1-SD increase in hemodynamic and arterial indexes.

**Table 3:** Multivariable-adjusted associations of ejection work density with blood pressure components and arterial properties

	Ejection work density (Pa)						
	All (*n* = 356)		Men (*n* = 197)		Women (*n* = 159)		
	Parameter estimate (95% CI)	*P* value	Parameter estimate (95% CI)	*P* value	Parameter estimate (95% CI)	*P* value	*P*_int_
*Conventional (brachial) BP*							
Systolic BP, + 16 mmHg	95.2 (76.8 to 113.6)	< 0.0001	82.8 (58.6 to 107.0)	< 0.0001	102.8 (74.7 to 130.9)	< 0.0001	0.074
Diastolic BP, + 9.5 mmHg	37.2 (17.0 to 57.4)	0.0003	28.0 (3.93 to 52.1)	0.023	52.3 (18.3 to 86.3)	0.0028	0.096
Pulse pressure, + 13 mmHg	82.1 (63.9 to 100.3)	< 0.0001	66.3 (43.2 to 89.4)	< 0.0001	94.2 (65.2 to 123.3)	< 0.0001	0.028
MAP, + 10 mmHg	69.6 (50.8 to 88.4)	< 0.0001	57.4 (33.6 to 81.1)	< 0.0001	81.9 (52.4 to 111.5)	< 0.0001	0.043
*SphygmoCor indexes*							
AP, + 9 mmHg	59.1 (31.5 to 86.6)	< 0.0001	74.3 (33.2 to 115.2)	0.0005	50.0 (12.3 to 87.7)	0.0098	0.42
Central PP, + 13 mmHg	65.7 (44.9 to 86.4)	< 0.0001	65.3 (37.4 to 93.2)	< 0.0001	62.5 (31.5 to 93.6)	0.0001	0.33
PWV, 1.9 m/s^a^	44.8 (19.5 to 70.1)	0.0006	39.1 (7.87 to 70.3)	0.015	44.0 (1.82 to 86.2)	0.041	0.47

The parameter estimates (95% confidence interval) indicate the change in EWD per 1 standard deviation increase in arterial index. All parameter estimates accounted for the variance explained by age, sex, heart rate and body height and weight. AP, central PP and PWV were additionally adjusted for MAP. P_int_ indicates the *P* values for an interaction between each arterial index and sex in predicting EWD. ^a^Data on arterial stiffness was available in 157 men and 117 women. *AP* indicates augmentation pressure, *BP* blood pressure, *MAP* mean arterial pressure, *PP* pulse pressure, *PWV* pulse wave velocity

As expected, in multivariable-adjusted analyses, higher EWD was independently related to higher systolic (effect size: + 95.2 Pa) and diastolic BP (+ 37.2 Pa; *P* ≤ 0.0003) as well as to higher brachial PP (+ 82.1 Pa) and mean arterial pressure (MAP; + 69.6 Pa; *P* < 0.0001). Moreover, after full adjustment, EWD increased with higher AP (+ 59.1 Pa), central PP (+ 65.7 Pa) and PWV (+ 44.8 Pa; *P* ≤ 0.0006; Table 3*;* Additional file 1: Figure S6). We also observed that EWD increased stronger with higher brachial PP and MAP in women than in men (Table 3*; P*_*int*_ ≤ 0.043).

We confirmed the associations between EWD and arterial characteristics in a sensitivity analysis excluding subjects on antihypertensive treatment (Additional file 1: Table S1). In addition, EWD standardized by RWT increased with all brachial and central BP components (*P* ≤ 0.011), but not with PWV (*P* = 0.13; Additional file 1: Table S2).

### Associations between EWD and LV structure and function

We determined the overall and sex-specific multivariable-adjusted estimates (95% CI) for EWD associated with a 1-SD increase in echocardiographic indexes reflecting LA and LV geometry (Table 4) and LV diastolic function (Table 5).

**Table 4:** Multivariable-adjusted associations between ejection work density and echocardiographic indexes of left atrial and left ventricular geometry

	Ejection work density (Pa)						
	All (*n* = 356)		Men (*n* = 197)		Women (*n* = 159)		
*LV and LA geometry*	Parameter estimate (95% CI)	*P* value	Parameter estimate (95% CI)	*P* value	Parameter estimate (95% CI)	*P* value	*P*_int_
*LV dimensions*							
Internal diameter, + 0.43 cm	10.7 (−14.2 to 35.6)	0.40	16.7 (−11.8 to 45.2)	0.25	10.0 (−34.9 to 55.0)	0.66	0.87
Septal wall, + 0.14 cm	−7.99 (−32.8 to 16.9)	0.53	− 23.9 (−51.4 to 3.62)	0.088	23.4 (−22.6 to 69.5)	0.32	0.0063
Posterior wall, + 0.12 cm	−14.8 (−40.2 to 10.6)	0.25	−34.3 (−61.1 to −7.42)	0.013	28.3 (− 22.5 to 79.1)	0.27	0.0014
RWT, + 0.05	−13.2 (− 34.3 to 7.85)	0.22	−29.9 (−53.1 to − 6.66)	0.012	12.3 (− 27.0 to 51.6)	0.54	0.0069
LV mass index, + 20 g/m^2^	6.06 (− 16.4 to 28.5)	0.60	−11.0 (−35.8 to 13.8)	0.38	46.1 (3.39 to 88.7)	0.035	0.0039
*Volumes*							
LA volume index, + 7.8 ml/m^2^	41.2 (19.2 to 63.2)	0.0003	23.9 (1.73 to 49.6)	0.067	76.2 (37.7 to 114.6)	0.0001	0.0019
EDV index, + 10 ml/m^2^	23.0 (1.18 to 44.8)	0.039	12.3 (−13.1 to 37.7)	0.34	43.8 (5.30 to 82.3)	0.026	0.14
ESV index, + 5 ml/m^2^	10.3 (−12.1 to 32.7)	0.37	−8.09 (−33.6 to 17.4)	0.53	39.8 (−0.90 to 80.5)	0.055	0.031
Stroke volume, + 15 ml	27.9 (3.49 to 52.4)	0.025	25.6 (−1.69 to 52.8)	0.066	37.8 (−8.22 to 83.9)	0.11	0.73
Ejection fraction, + 5.1%	11.5 (−7.26 to 30.2)	0.23	23.9 (1.16 to 46.6)	0.040	1.19 (−30.1 to 32.4)	0.94	0.13

The parameter estimates (95% confidence interval) indicate the change in EWD per 1 standard deviation increase in the echocardiographic index. All parameter estimates accounted for the variance explained by age, sex, heart rate and body height and weight. P_int_ represents the *P* value for an interaction between each LV index and sex in predicting EWD. Adjustment for BSA-indexed measures did not include body height and weight. *BSA* indicates body surface area, *EDV* end-diastolic volume, *ESV* end-systolic volume, *LA* left atrial, *PWT* posterior wall thickness

**Table 5:** Multivariable-adjusted associations between ejection work density and echocardiographic indexes of left ventricular diastolic function

	Ejection work density (Pa)						
	All (*n* = 356)		Men (*n* = 197)		Women (*n* = 159)		
*LV diastolic function index*	Parameter estimate (95% CI)	*P* value	Parameter estimate (95% CI)	*P* value	Parameter estimate (95% CI)	*P* value	*P*_int_
E peak, + 15 cm/s	55.3 (33.0 to 77.5)	< 0.0001	38.8 (11.3 to 66.3)	0.0059	86.7 (51.2 to 122.3)	< 0.0001	0.39
A peak, + 15 cm/s	47.2 (20.6 to 73.8)	0.0005	43.9 (9.50 to 78.2)	0.013	45.5 (39.0 to 87.2)	0.032	0.16
E/A ratio, + 0.45	29.1 (−2.30 to 60.4)	0.069	17.0 (−20.9 to 54.9)	0.38	58.3 (5.50 to 111.1)	0.031	0.39
e’ peak, + 3.2 cm/s	32.2 (−0.031 to 64.3)	0.050	48.6 (10.3 to 87.0)	0.013	16.5 (−36.7 to 72.7)	0.56	0.014
a’ peak, + 2.0 cm/s	19.7 (−4.97 to 44.4)	0.12	33.9 (5.42 to 52.3)	0.020	−8.96 (−51.9 to 34.0)	0.68	0.75
E/e’ ratio, + 2.1	32.1 (9.20 to 55.1)	0.0062	2.45 (−31.1 to 36.1)	0.89	51.1 (19.6 to 82.6)	0.0017	0.0016

The parameter estimates (95% confidence interval) indicate the change in EWD per 1 standard deviation increase in the LV diastolic function index. All parameter estimates accounted for the variance explained by age, sex, heart rate and body height and weight. P_int_ represents the *P* value for an interaction between each LV diastolic function index and sex in predicting EWD

#### EWD in relation to LA and LV geometry

**Fig. 4:**
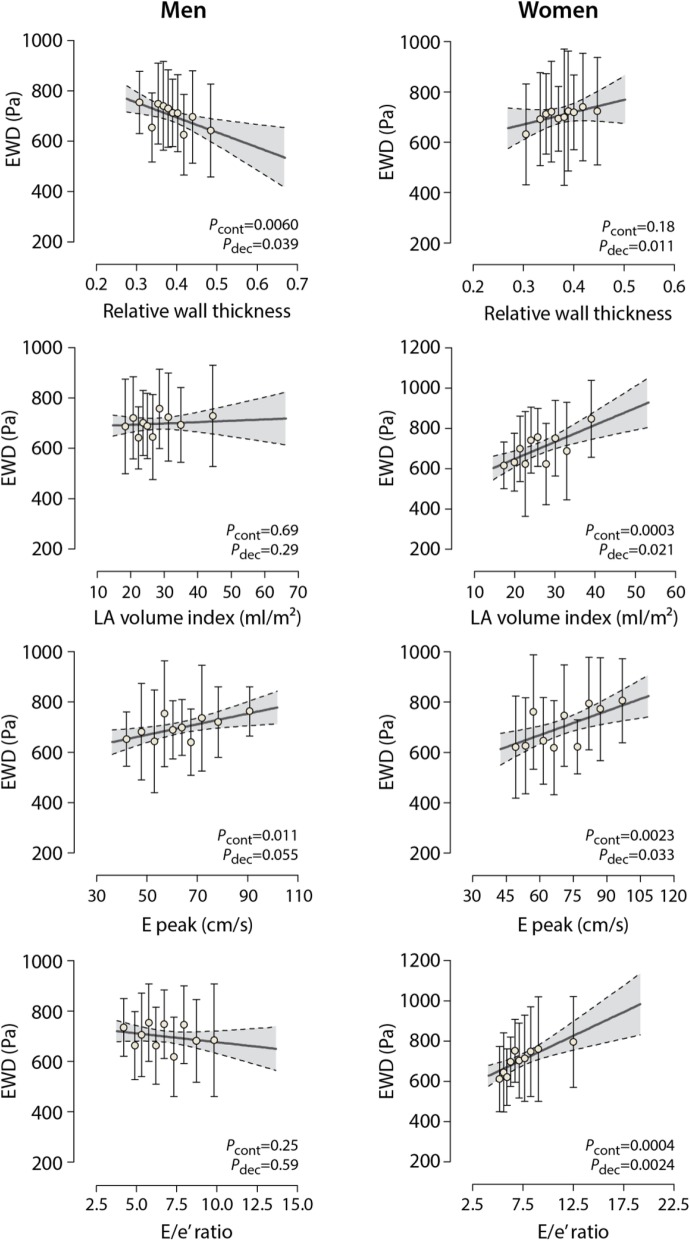
Multivariable-Adjusted Ejection Work Density (EWD) by Deciles of LA and LV Indexes. Data markers are centered on the means in each decile. Analysis was adjusted as described in Tables 4 and 5 legends. Full line and shaded area represent the regression line and 95% confidence interval for the change in EWD and LV index on a continuous scale. *P* values are for linear trend between EWD and LV index on a continuous (*P*_cont_) or decile scale (*P*_dec_)

In all subjects, EWD significantly increased with LAVi (+ 41.2 Pa), end-diastolic volume index (EDVi; + 23.0 Pa) and LV stroke volume (+ 27.9 Pa) after full adjustment (*P* ≤ 0.039 for all; Table 4). EWD decreased significantly with increased LV posterior wall thickness (− 34.3 Pa) and RWT (− 29.9 Pa) only in men (*P* ≤ 0.013; Table 4; Fig. 4). In contrast, EWD increased significantly with higher LV mass (+ 46.1 Pa), LAVi (+ 76.2 Pa) and EDVi (+ 43.8 Pa) only in women (*P* ≤ 0.035; Table 4*;* Fig. 4).

We confirmed these associations when excluding subjects receiving antihypertensive drug therapy (Additional file 1: Table S3).

Similarly, when standardizing by RWT, EWD increased with higher LAVi, EDVi and end-systolic volume index (ESVi) and stroke volume (*P* ≤ 0.012; Additional file 1: Table S4).

#### EWD in relation to LV function

Elevated EWD correlated independently to greater early (+ 55.3 Pa) and late (+ 47.2 Pa) diastolic transmitral peak velocities (*P* ≤ 0.0005; Table 5). After full adjustment, EWD related directly with the E/e’ ratio (+ 32.1 Pa; *P* = 0.0062; Table 5). However, in sex-specific analyses, this relationship was only observed in women (Fig. 4). EWD correlated directly with peak global LS in both men and women (+ 65.4 Pa; *P* < 0.0001).

We confirmed these associations when excluding subjects on antihypertensive drug treatment (Additional file 1: Table S5). Standardized by RWT, EWD increased independently with transmitral velocities, E/A ratio and e’ peak (*P* ≤ 0.027 for all; Additional file 1: Table S4).

## Discussion

Here, we investigated the anthropometric and clinical determinants of a novel index of LV myocardial performance as derived from non-invasive, simultaneous pressure-strain recordings. In addition, we explored its relation to arterial stiffness and LV function and structure. The key findings of our study were as follows: (i) older age, female sex and hypertensive status are associated with higher EWD; (ii) in both men and women, EWD increased with steady and pulsatile BP components and arterial stiffness; and (iii) EWD decreased independently with LV wall thickness in men, yet increased with higher LAVi and LV filling pressure (E/e’ ratio) in women only.

LV performance is determined by its intrinsic contractility (inotropy), by the tension on the LV myocardium at end-diastole (preload) and by the load against which the LV needs to contract to eject blood (afterload) [1]. Within this context, the work density at which the LV generates a stroke volume against a given afterload might be represented by the area of the LV pressure-strain loop during ejection (i.e. EWD). Indeed, EWD integrates the instantaneous LV deformation against an instantaneous pressure and might thus reflect LV systolic performance [2, 11].

Numerous population studies demonstrated the detrimental impact of a chronically increased afterload on LV structure and function [4–7]. Indeed, at increased afterload, the heart needs to generate greater force to preserve stroke volume, thereby increasing its energy expenditure and, eventually, promoting cardiac dysfunction and remodeling. Along these lines, an experimental study showed that the peak rate of changes in LV pressure (dP/dt), an invasive index of contractility, was 51% greater in hypertensive than in normotensive rabbits [17]. We previously observed that the higher arterial load in subjects with hypertension matched with higher LV systolic stiffness and enhanced LV myocardial performance [2]. In result, EWD was 24% higher in hypertensive subjects as compared to normotensives [2]. In line with a small case-control study in 74 patients [18], we confirmed that hypertensive subjects had a higher pressure-strain area than normotensive participants if untreated or if their hypertension was uncontrolled, even after accounting for important confounders such as age, sex and body size. In contrast, EWD was not different between normotensives and effectively controlled hypertensives. Evidently, the observational and cross-sectional nature of our study did not allow to infer true causality between controlled hypertension and normalization of EWD. Moreover, EWD increased with PWV, reflecting aortic stiffness.

Previous population studies demonstrated that LV diastolic function, particularly in women, is sensitive to the detrimental effects of increased central pulse pressure and arterial stiffness [4, 6, 7]. This sex-dependent vulnerability of LV diastolic function to cardiac afterload especially in older women might be explained by the higher aortic pulsatile load and stiffness [5, 7] and the enhanced LV systolic performance [9, 19] in women as compared to men. Indeed, women have on average smaller heart chamber volumes, even after standardization for body size [19], with higher LV ejection fraction and global LS [15], and a steeper slope of the stroke work-EDV relationship [19]. Of note, the passive LV diastolic elastance, a major determinant of LV filling pressure, also appears higher in women than in men [19]. In line, we observed that women had higher pulsatile load and LV LS and, therefore, overall higher EWD than men. Moreover, EWD increased significantly with echocardiographic surrogates of LV filling pressure (E/e’ ratio) [20] and preload indexes like LAVi and EDVi in women only [21]. As such, EWD might reflect the sex-dependent interplay between preload, afterload and LV systolic performance.

Besides undergoing changes in function, the LV progressively remodels in response to chronically elevated LV afterload [4, 22]. Indeed, the LV walls thicken during chronic pressure overload in order to normalize LV wall stress [22]. In fact, the LV tends towards concentric remodeling to cope with the progressive stiffening of the large arteries during life [4]. In our study, yet only observed in men, EWD decreased with LV concentric remodeling (higher RWT).

Recent interest in constructing and evaluating pressure-strain loops supports the need for a non-invasive clinical tool that integrates LV deformation and loading. Indeed, inspired by Russel and colleagues [23], GE Healthcare recently added a feature to their echocardiographic post-processing software (EchoPAC) to construct pressure-strain loops from 2D LV strain and an estimated LV pressure curve. The LV pressure-strain loop area derived from such estimated loops correlated strongly with invasive measurements [12]. Yet, Hubert et al. observed substantial shortcomings in the estimation of the pressure curve, particularly at greater pressures and loop areas [12]. In contrast, in our study, we utilized the true area of subject-specific pressure waveforms at the specific time of strain recording. As such, our approach does not only account for differences in the shape of pressure waves between individuals, but also for temporal variability in pressure magnitude within individuals. However, validation of our approach to invasive methods is required.

The present study must be interpreted in context of its limitations and strengths. First, echocardiography is prone to measurement errors. However, one experienced observer recorded all echocardiographic images using a standardized protocol. Moreover, images were post-processed by two experienced observers with good reproducibility. Second, we derived EWD by simultaneous finger tonometry and 2D speckle tracking to overcome the technical challenges linked to simultaneous carotid tonometry and Tissue Doppler Imaging [2]. Of notice, EWD values derived from our 356 participants corresponded to those obtained by carotid tonometry and TDI in a random cohort of 148 participants [2]. Finally, EWD requires further validation in outcome and experimental studies.

## Conclusion

Older age, female sex and hypertensive status are associated with higher EWD. Integration of the LV pressure-strain loop during ejection might be a useful tool to non-invasively evaluate sex-specific and interdependent effects of preload and afterload on LV myocardial performance. Future studies should investigate the utility of pressure-strain loops in clinical decision-making, particularly in subjects at risk for heart failure.

## Additional file

Additional file 1: Area of the pressure-strain loop during ejection as non-invasive index of left ventricular performance: supplemental material. (DOCX 814 kb)

## Data Availability

The datasets used and/or analyzed during the current study are available from the corresponding author (T.K.) upon reasonable request.

## Abbreviations

AP: Augmentation pressure
EDVi: End-diastolic volume index
ESVi: End-systolic volume index
EWD: Ejection work density
FLEMENGHO: Flemish Study on Environment, Genes and Health Outcomes
LAVi: Left atrial volume index
LS: Longitudinal strain
LVMi: Left ventricular mass index
PP: Pulse pressure
PWV: Pulse wave velocity
