# Supplementary material for: Area of the pressure-strain loop during ejection as non-invasive index of left ventricular performance: a population study
Source: Cardiovasc Ultrasound. 2019 Aug 5;17:15. doi: 10.1186/s12947-019-0166-y (PMC6683340; doi:10.1186/s12947-019-0166-y)
Supplement: Supplementary file 1 — Area of the pressure-strain loop during ejection as non-invasive index of left ventricular performance: supplemental material. (DOCX 814 kb) [file 12947_2019_166_MOESM1_ESM.docx]

**Area of the pressure-strain loop during ejection as non-invasive index of left ventricular performance: a population study**

SUPPLEMENTAL DATA

**ECHOCARDIOGRAPHY.** All subjects refrained from smoking, heavy exercise and drinking alcohol or caffeinated beverages for at least 2 hours prior to the examination. To ensure steady state, echocardiography and arterial phenotyping were performed after the subjects had rested for at least 15 min in the supine position.

*Conventional echocardiogram -* One experienced physician (T.K.) did the ultrasound examination using a Vivid E9 (GE Vingmed, Horten, Norway) interfaced with a 2.5- to 3.5-MHz phased-array probe, in accordance to recommendations (1) and as described previously (2). With the subjects in partial left decubitus and breathing normally, the observer obtained images along the parasternal long and short axes and along the apical 4- and 2-chamber long-axis views, together with a simultaneous electrocardiogram signal. All digital recordings lasted 5 cardiac cycles. M-mode tracings were recorded from the LV parasternal long-axis view under guidance of the 2D image. The ultrasound beam was positioned just below the mitral valve at the level of the posterior chordae tendineae. To record pulsed wave transmitral Doppler waveforms from the apical window, the observer positioned a 1- to 3-mm Doppler sample volume at the mitral valve tips. Using Tissue Doppler Imaging (TDI), the observer recorded low-velocity, high-intensity myocardial signals at a high frame rate (>190 FPS), while ensuring a parallel alignment of the ultrasound beam with the myocardial segment of interest. The sonographer placed a 5-mm pulsed Doppler sample at the septal, lateral, inferior and posterior sites of the mitral annulus to record mitral annulus velocities from the apical window.

*Off-line analysis -* Blinded to the participants’ characteristics, one experienced observer (TK) analyzed the conventional echocardiograms using EchoPac software (GE Vingmed). Measurements of at least 3 different heart cycles were averaged for statistical analysis. LV internal diameter and interventricular septal and posterior wall thickness were measured from 2D-guided M-mode tracings at end-diastole. When optimal orientation of M-mode ultrasound beam could not be obtained, the reader performed linear measurements on correctly oriented two-dimensional images. End-diastolic LV dimensions were used to calculate LV mass using an anatomically validated formula. Relative wall thickness (RWT) was calculated as 0.5 x (interventricular septum + posterior wall) / LV internal diameter at end-diastole. Using the standard Simpson method, LV end-diastolic volume (EDV), end-systolic volume (ESV) and ejection fraction (EF) were derived from the apical 4- and 2 chamber views. LV mass and LV and left atrial volumes were indexed to body surface area (BSA). Transmitral blood flow Doppler signals were used to measure peak early (E) and late (A) diastolic velocities as well as their ratio (E/A). From pulsed-wave TDI recordings, we measured the systolic (s') and early (e') and late (a’) diastolic peak velocities of the mitral annulus displacement at 4 acquisition sites (septal, lateral, inferior and posterior). We calculated the E/e' ratio, a surrogate of LV filling pressure, by dividing transmitral E peak by e' averaged from the 4 acquisition sites.

As reported before (3), an experienced physician (T.K.) analyzed the conventional echocardiograms of 17 subjects twice to determine intra-observer reproducibility. Intra-observer reproducibility coefficient of a measurement was the 2SD interval about the mean of the relative differences across pairwise readings. The intra-observer reproducibility was 2.2% for LV internal end-diastolic diameter, 4.6% for LV wall thickness and 4.3% for LV mass, whereas the intra-observer reproducibility for the tissue Doppler velocities ranged from 4.5% to 5.3% for e’ velocities and from 4.0% to 4.5% for a’ velocities across the four sampling sites.

Two experienced observers (N.C and T.K.) measured LV global longitudinal strain (LS) using myocardial speckle-tracking software (Q-analysis, GE Vingmed) at default settings. The LV endocardial border was manually traced at the end-systolic frame of the two-dimensional 4 and 2 chamber views. The software automatically tracked myocardial speckle motion while dividing the region of interest in LV basal, mid and apical levels. We adjusted the region of interest after visual evaluation of the tracking. Images were rejected if tracking was inadequate in ≥2 segments per view. We used absolute values of peak systolic, mid-wall global LS for statistical analysis. Relative intra- and interobserver reproducibility of global LS was 6.1% and 7.3%, respectively (4).

*Finger applanation tonometry* - During the echocardiographic examination, we performed finger applanation tonometry using a Finometer Pro device (Finapres Medical Systems, Amsterdam, The Netherlands), which non-invasively recorded continuous finger pressure waves at the subject’s right middle-finger (5). Calibrated digital pulses were converted to brachial pressure curves by use of a validated transfer function implemented in the Finometer software (6). Finapres recordings were discarded when diastolic BP was less than 50 mmHg.

**ARTERIAL MEASUREMENTS.** Brachial (peripheral) BP was the average of three consecutive readings obtained in supine position by the validated OMRON 705CP oscillometric sphygmomanometer (Omron Inc., Kyoto, Japan). A standard cuff of 22x12 cm with an inflatable bladder was used for subjects with an arm circumference of less than 32 cm. For greater arm circumferences, a cuff with a 35x15 cm bladder was used.

We recorded carotid, femoral and radial arterial waveforms by applanation tonometry during an 8-second period, using a high-fidelity SPC-301 micromanometer (Millar Instruments, Inc., Houston, TX, USA) interfaced with a computer running SphygmoCor software version 7.1 (AtCor Medical Pty. Ltd., West Ryde, New South Wales, Australia). Recordings were discarded when the systolic or diastolic variability of consecutive waveforms exceeded 5% or when the amplitude of the pulse wave signal was less than 80 mV. The pulse waves were calibrated by the supine brachial BP, which, as described above, was measured immediately before the tonometric recordings. From the radial signal, the SphygmoCor software constructed the aortic pulse wave by means of a validated generalized transfer function. The software returned the central systolic BP and the pressure at the first (P1) and second (P2) peak or shoulder of the central waveform. Central pulse pressure (PP) was calculated as the difference between central systolic and diastolic pressure. Augmentation pressure (AP) was the difference between P2 and P1 of the aortic pulse wave. In a subset of 157 men and 117 women, we measured aortic pulse wave velocity (PWV), the current non-invasive gold standard of arterial stiffness (7). PWV was measured by sequential electrocardiographically-gated recordings of the arterial pressure waveform at the carotid and femoral arteries. We measured the distance from the suprasternal notch to the carotid sampling site and from the suprasternal notch to the femoral sampling site. Pulse transit time was the average of 10 consecutive beats. PWV was the ratio of the carotid-femoral distance (in meters) to the carotid-femoral transit time of the pressure wave (in seconds). The intra-observer intra-session reproducibility of carotid-femoral PWV was 2.61% (8).

**OTHER MEASUREMENTS.** We administered standardized questionnaires for detailed information on the subject’s medical history, smoking and drinking habits and intake of medications. Conventional blood pressure was the average of 5 auscultatory readings obtained with the subject in seated position. Hypertension was defined as a blood pressure of at least 140 mm Hg systolic or 90 mm Hg diastolic or the use of antihypertensive drugs. Body mass index was weight in kilograms divided by the square of height in meters. Diabetes mellitus was determined by self-report, a fasting glucose level of at least 126 mg/dL, or the use of antidiabetic agents.

**REFERENCES**

1. Gottdiener JS, Bednarz J, Devereux RB, et al. American Society of Echocardiography recommendations for use of echocardiography in clinical trials. J Am Soc Echocardiogr. 2004;17:1086–119.

2. Kuznetsova T, Herbots L, Lopez B, et al. Prevalence of left ventricular diastolic dysfunction in a general population. Circ. Heart Fail. 2009;2:105–112.

3. Cauwenberghs N, Knez J, Tikhonoff V, et al. Doppler indexes of left ventricular systolic and diastolic function in relation to the arterial stiffness in a general population. J. Hypertens. 2016;34:762–771.

4. Kuznetsova T, Cauwenberghs N, Knez J, et al. Additive Prognostic Value of Left Ventricular Systolic Dysfunction in a Population-Based Cohort. Circ. Cardiovasc. Imaging 2016;9.

5. Schutte AE, Huisman HW, Van Rooyen JM, Oosthuizen W, Jerling JC. Sensitivity of the Finometer device in detecting acute and medium-term changes in cardiovascular function. Blood Press. Monit. 2003;8:195–201.

6. Schutte AE, Huisman HW, van Rooyen JM, Malan NT, Schutte R. Validation of the Finometer device for measurement of blood pressure in black women. J. Hum. Hypertens. 2004;18:79–84.

7. Vlachopoulos C, Xaplanteris P, Aboyans V, et al. The role of vascular biomarkers for primary and secondary prevention. A position paper from the European Society of Cardiology Working Group on peripheral circulation: Endorsed by the Association for Research into Arterial Structure and Physiology (ARTERY. Atherosclerosis 2015;241:507–532.

8. Liu Y-P, Thijs L, Kuznetsova T, et al. Central systolic augmentation indexes and urinary sodium in a white population. Am. J. Hypertens. 2013;26:95–103.


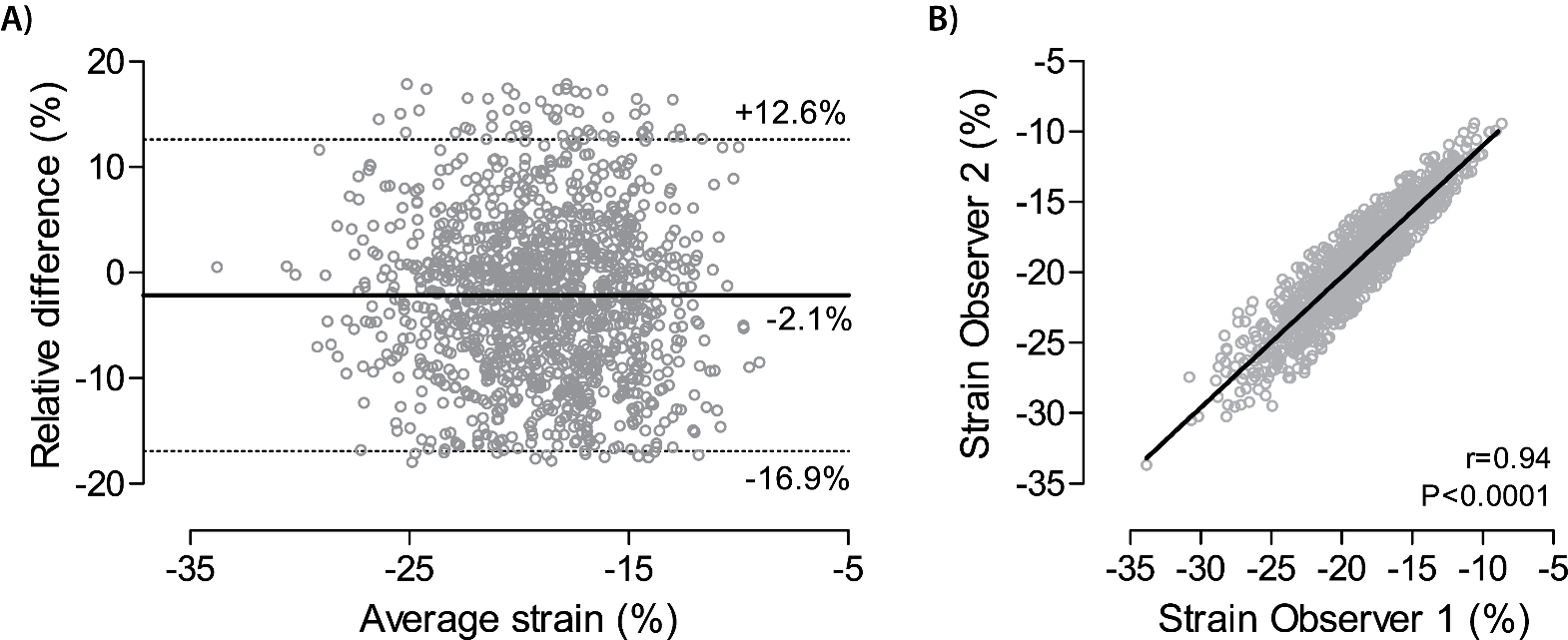
FIGURE S1. **Interobserver reproducibility of left ventricular strain.** (A) Bland-Altman plot representing the relative interobserver variability of segmental LS values. Biases and 95% limits of agreement are shown as full and dotted lines, respectively. (B) Correlation between the pairwise measurements of segmental LS by both observers. LS indicates longitudinal strain.


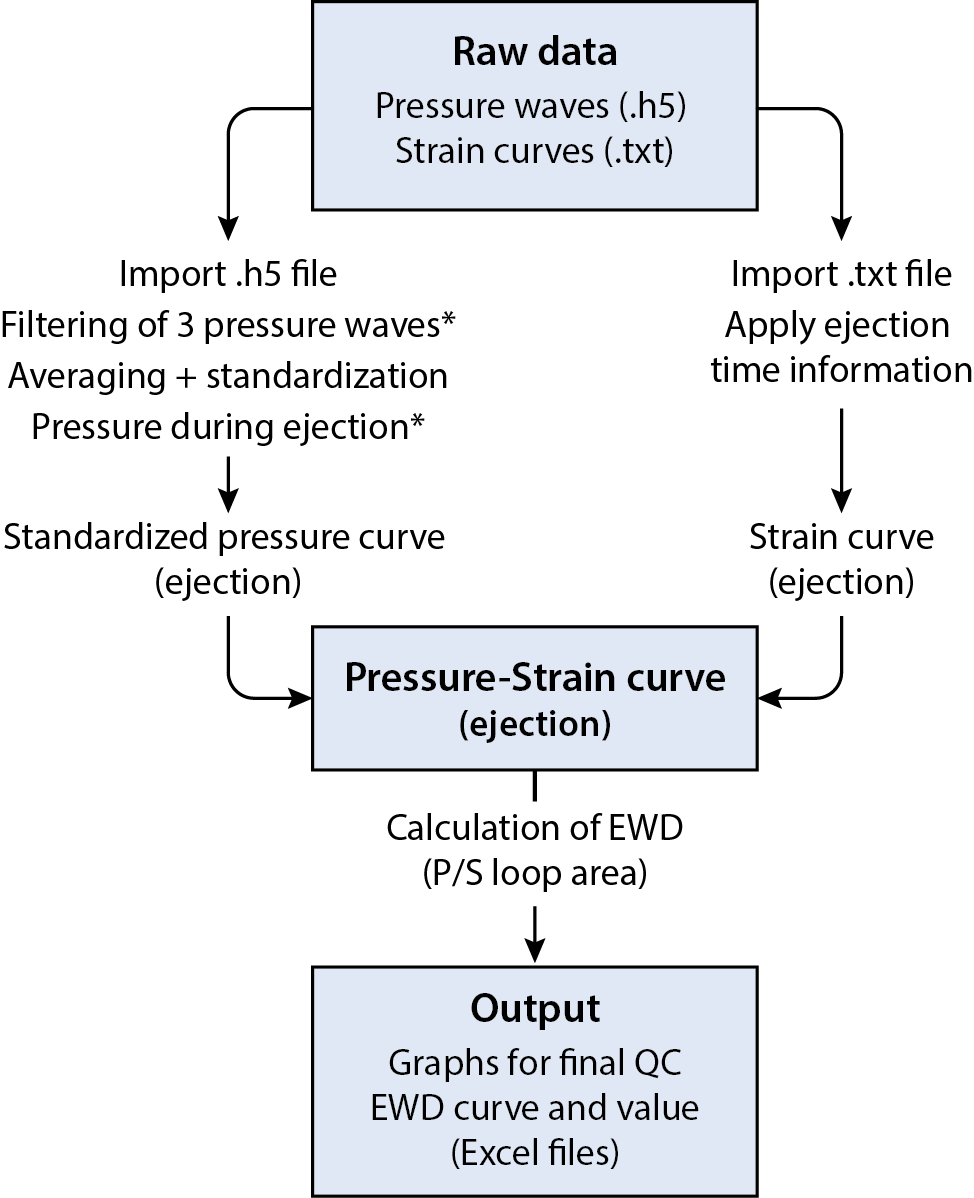
FIGURE S2. **Custom Matlab Algorithm to Derive the Ejection Work Density (EWD) from Simultaneous LV Strain and Pressure Recordings**. We embedded intermediate (*) and final check points for quality control (QC). Final QC includes visual evaluation of graphically presented pressure, LV strain and pressure-strain curves.

FIGURE S3. **Distributions of the Ejection Work Density (EWD) by echocardiographic view.** EWD from 4 chamber (left) and 2 chamber (middle) echocardiographic views were averaged to retrieve the overall EWD (right). The full line represents the fitted normal density plot.


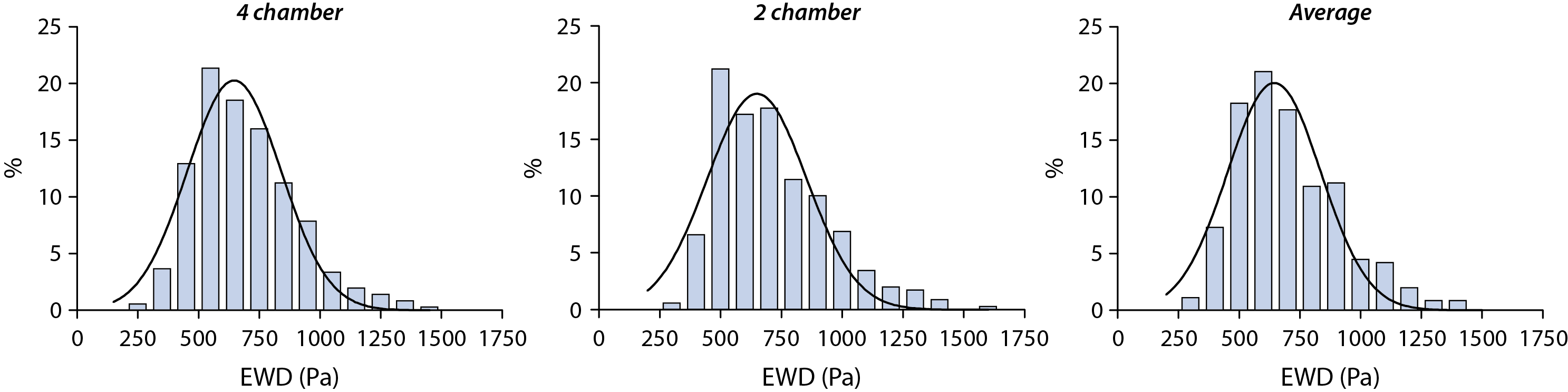


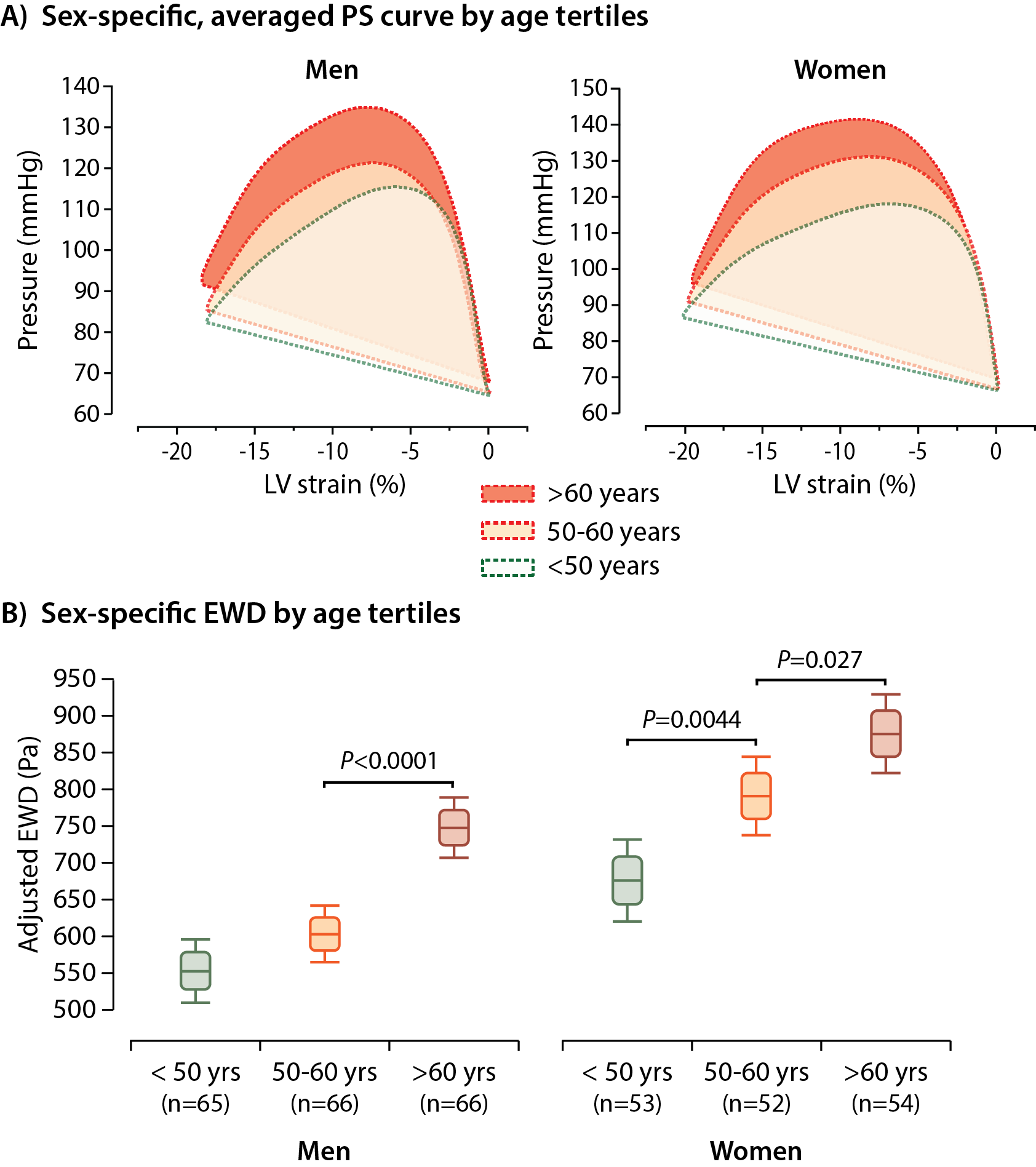
FIGURE S4. **The Ejection Work Density (EWD) in Relation to Age by Sex.** (A) Sex-specific, average pressure-strain (PS) curves by age tertiles. (B) Boxplots present sex-specific, mean EWD and 5-95% and 25-75% confidence limits adjusted for heart rate and body height and weight by age tertiles.

**
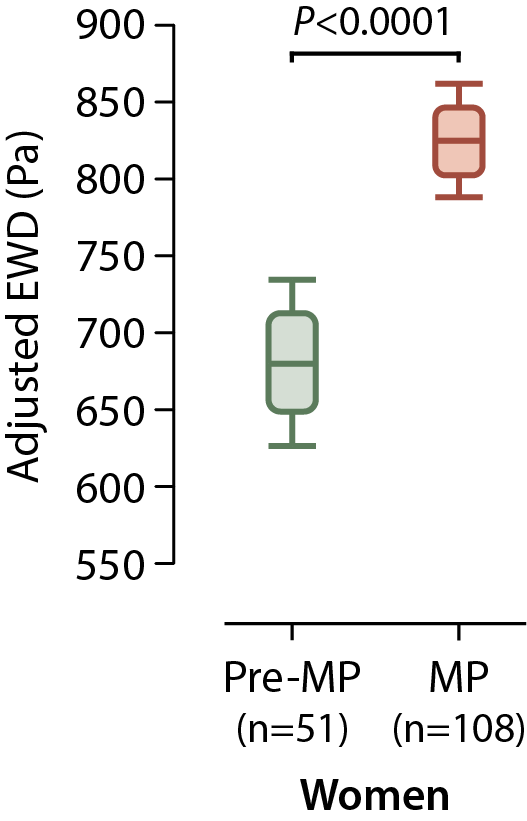
**FIGURE S5. **The Ejection Work Density (EWD) in Relation to Menopausal State.** Boxplot presents the mean EWD and 5-95% and 25-75% confidence limits adjusted for heart rate and body height and weight.


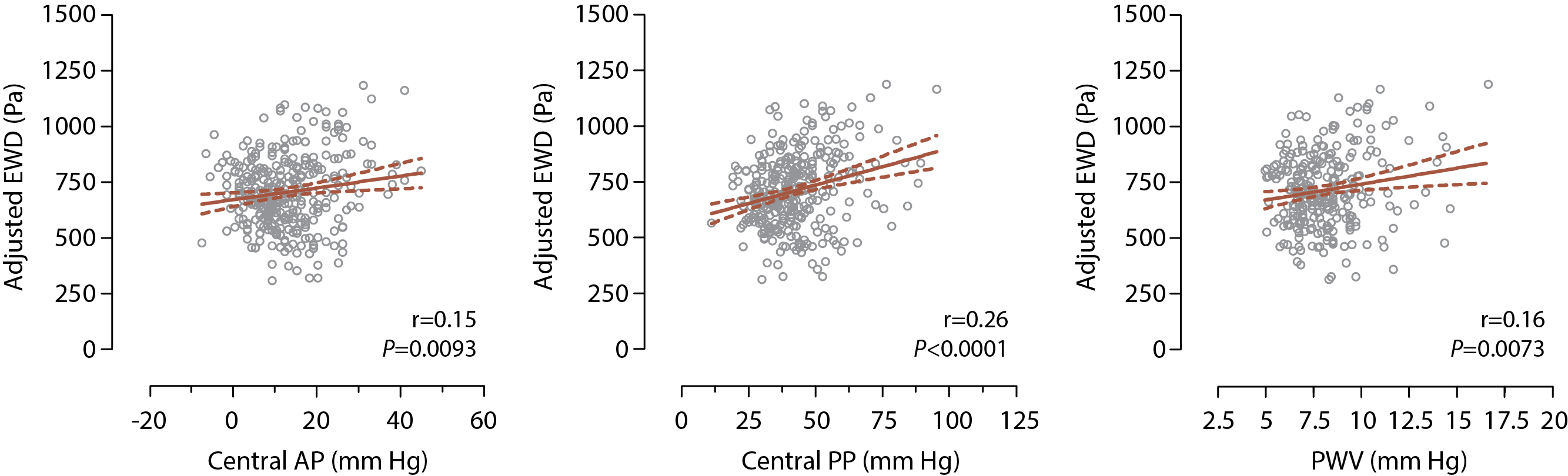
Figure S6. **Multivariable-Adjusted Relation between Ejection Work Density (EWD) and Arterial Properties.** EWD was adjusted for age, sex, heart rate, MAP, body height and body weight. Full and dotted line represent the regression line and 95% confidence band, respectively. AP indicates augmentation pressure; MAP, mean arterial pressure; PP, pulse pressure; PWV, pulse wave velocity.

TABLE S1. **Multivariable-Adjusted Associations between Ejection Work Density and Arterial Characteristics** **in 264 Subjects not Receiving Anti-Hypertensive Treatment.**

|  | Ejection Work Density (Pa) | | | | | |  |
| --- | --- | --- | --- | --- | --- | --- | --- |
|  | All (n=264) | | Men (n=143) | | Women (n=121) | |  |
|  | Parameter estimate (95% CI) | *P* value | Parameter estimate (95% CI) | *P* value | Parameter estimate (95% CI) | *P* value | *P*_int_ |
| *Conventional (brachial) BP* |  |  |  |  |  |  |  |
| Systolic BP, +16 mm Hg | 100.8 (78.8 to 122.8) | <0.0001 | 89.5 (59.5 to 119.5) | <0.0001 | 100.0 (66.9 to 133.1) | <0.0001 | 0.16 |
| Diastolic BP, +9.5 mm Hg | 26.0 (2.81 to 49.2) | 0.028 | 24.4 (-1.20 to 49.9) | 0.062 | 2.68 (-14.4 to 68.0) | 0.20 | 0.26 |
| Pulse pressure, +13 mm Hg | 88.8 (67.9 to 109.6) | <0.0001 | 62.8 (35.1 to 90.5) | <0.0001 | 101.1 (69.2 to 133.0) | <0.0001 | 0.016 |
| MAP, + 10 mm Hg | 64.3 (42.0 to 86.6) | <0.0001 | 53.4 (26.3 to 80.5) | 0.0002 | 68.7 (32.6 to 104.8) | 0.0003 | 0.066 |
| *Arterial properties* |  |  |  |  |  |  |  |
| AP, + 9 mm Hg | 49.7 (17.7 to 81.6) | 0.0024 | 73.7 (24.5 to 122.8) | 0.0036 | 37.1 (-6.17 to 80.4) | 0.092 | 0.28 |
| Central PP, +13 mmHg | 67.6 (43.5 to 91.6) | <0.0001 | 71.7 (38.5 to 105.0) | <0.0001 | 59.4 (23.9 to 94.9) | 0.0013 | 0.64 |
| PWV, 1.9 m/s* | 59.5 (30.0 to 88.9) | <0.0001 | 45.7 (6.26 to 85.1) | 0.024 | 60.7 (14.6 to 106.8) | 0.011 | 0.28 |

Parameter estimates (95% confidence interval) indicate changes in ejection work density associated with a 1 standard deviation increase in arterial index. All parameter estimates were adjusted for age, sex, heart rate and body height and weight. AP, central PP and PWV were additionally adjusted for MAP. P_int_ indicates the P values for the interaction of conventional BP components and arterial properties with sex. *Data on arterial stiffness was available in 116 men and 91 women. AP indicates augmentation pressure; BP, blood pressure; MAP, mean arterial pressure; PP, pulse pressure; PWV, pulse wave velocity.

TABLE S2. **Multivariable-Adjusted Associations between Ejection Work Density Standardized to Relative Wall Thickness and Arterial Characteristics.**

|  | Ejection Work Density / RWT (Pa) | | | | | |  |
| --- | --- | --- | --- | --- | --- | --- | --- |
|  | All (n=356) | | Men (n=197) | | Women (n=159) | |  |
|  | Parameter estimate (95% CI) | *P* value | Parameter estimate (95% CI) | *P* value | Parameter estimate (95% CI) | *P* value | *P*_int_ |
| *Conventional (brachial) BP* |  |  |  |  |  |  |  |
| Systolic BP, +16 mm Hg | 230.0 (174.2 to 285.7) | <0.0001 | 191.1 (115.7 to 267.2) | <0.0001 | 259.6 (176.2 to 343.1) | <0.0001 | 0.12 |
| Diastolic BP, +9.5 mm Hg | 76.9 (17.5 to 136.2) | 0.011 | 52.5 (-20.2 to 125.2) | 0.16 | 116.0 (17.1 to 215.0) | 0.022 | 0.18 |
| Pulse pressure, +13 mm Hg | 207.4 (153.0 to 261.8) | <0.0001 | 162.6 (91.7 to 233.6) | <0.0001 | 248.6 (163.3 to 334.0) | <0.0001 | 0.058 |
| MAP, + 10 mm Hg | 160.3 (104.1 to 216.5) | <0.0001 | 124.4 (51.3 to 197.4) | 0.0010 | 198.5 (111.3 to 285.8) | <0.0001 | 0.085 |
| *Arterial properties* |  |  |  |  |  |  |  |
| AP, + 9 mm Hg | 185.0 (102.2 to 267.8) | <0.0001 | 247.4 (120.9 to 373.9) | 0.0002 | 146.2 (34.0 to 258.4) | 0.011 | 0.82 |
| Central PP, +13 mmHg | 171.9 (108.4 to 235.4) | <0.0001 | 171.4 (83.6 to 259.2) | 0.0002 | 164.6 (71.3 to 257.9) | 0.0007 | 0.49 |
| PWV, 1.9 m/s* | 59.3 (-17.7 to 136.4) | 0.13 | 47.3 (-49.3 to 143.8) | 0.33 | 47.4 (-79.4 to 174.0) | 0.46 | 0.59 |

Parameter estimates (95% confidence interval) indicate changes in ejection work density associated with a 1 standard deviation increase in arterial index. All parameter estimates were adjusted for age, sex, heart rate and body height and weight. AP, central PP and PWV were additionally adjusted for MAP. P_int_ indicates the P values for the interaction of conventional BP components and arterial properties with sex. *Data on arterial stiffness was available in 157 men and 117 women. AP indicates augmentation pressure; BP, blood pressure; MAP, mean arterial pressure; PP, pulse pressure; PWV, pulse wave velocity.

TABLE S3. **Multivariable-Adjusted Associations between Ejection Work Density and Echocardiographic Indexes of Left Atrial and Left Ventricular Geometry in 264 Subjects not Receiving Anti-Hypertensive Treatment.**

|  | Ejection work density (Pa) | | | | | |  |
| --- | --- | --- | --- | --- | --- | --- | --- |
|  | All (n=264) | | Men (n=143) | | Women (n=121) | |  |
| *LA and LV geometry* | Parameter estimate (95% CI) | *P* value | Parameter estimate (95% CI) | *P* value | Parameter estimate (95% CI) | *P* value | *P*_int_ |
| *LV dimensions* |  |  |  |  |  |  |  |
| Internal diameter, +0.43 cm | 14.2 (-14.7 to 43.1) | 0.33 | 25.7 (-6.84 to 58.3) | 0.12 | 3.80 (-45.5 to 53.1) | 0.88 | 0.44 |
| Septal wall, +0.14 cm | -15.6 (-44.0 to 12.9) | 0.28 | -21.4 (-51.8 to 89.1) | 0.16 | -4.71 (-56.8 to 47.4) | 0.86 | 0.070 |
| Posterior wall, +0.12 cm | -14.8 (-44.8 to 15.1) | 0.33 | -25.7 (-56.3 to 4.78) | 0.098 | -1.02 (-58.4 to 60.4) | 0.97 | 0.035 |
| RWT, +0.05 | -17.1 (-41.0 to 6.82) | 0.16 | -30.0 (-56.3 to -3.58) | 0.026 | -4.20 (-45.9 to 37.5) | 0.84 | 0.025 |
| LV mass index, +20 g/m² | 11.7 (-16.5 to 39.9) | 0.41 | 1.09 (-28.6 to 30.8) | 0.94 | 35.7 (-17.8 to 89.4) | 0.19 | 0.056 |
| *Volumes* |  |  |  |  |  |  |  |
| LA volume index, +7.8 ml/m² | 56.0 (27.8 to 84.2) | 0.0001 | 52.0 (18.2 to 85.7) | 0.0028 | 65.3 (19.8 to 110.8) | 0.0053 | 0.11 |
| EDV index, +10 ml/m² | 21.9 (-2.89 to 46.7) | 0.083 | -3.23 (-30.8 to 24.3) | 0.82 | 60.0 (16.4 to 103.5) | 0.0074 | 0.013 |
| ESV index, +5 ml/m² | 10.5 (-14.4 to 35.3) | 0.41 | -22.5 (-49.3 to 4.26) | 0.099 | 53.5 (8.82 to 98.2) | 0.019 | 0.0023 |
| Stroke volume, +15 ml | 22.7 (-4.94 to 50.4) | 0.063 | 11.1 (-18.2 to 40.4) | 0.23 | 55.4 (2.89 to 107.8) | 0.039 | 0.35 |
| Ejection fraction, +5.1% | 5.95 (-14.6 to 26.5) | 0.57 | 22.5 (-1.25 to 46.2) | 0.063 | 2.00 (-32.3 to 36.4) | 0.91 | 0.23 |

Parameter estimates (95% confidence interval) indicate changes in ejection work density associated with a 1 standard deviation increase in arterial index. All parameter estimates were adjusted for age, sex, heart rate and body height and weight. Adjustment for BSA-indexed measures did not include body height and weight. P_int_ indicates the P values for the interaction between echocardiographic indexes and sex. BSA indicates body surface area; EDV, end-diastolic volume; ESV, end-systolic volume; LA, left atrial; LV, left ventricular; RWT, relative wall thickness.

TABLE S4. **Multivariable-Adjusted Associations between Ejection Work Density Standardized to Relative Wall Thickness and Echocardiographic Indexes of Left Atrial and Left Ventricular Geometry.**

|  | Ejection Work Density / RWT (Pa) | | | | | |  |
| --- | --- | --- | --- | --- | --- | --- | --- |
|  | All (n=356) | | Men (n=197) | | Women (n=159) | |  |
|  | Parameter estimate (95% CI) | *P* value | Parameter estimate (95% CI) | *P* value | Parameter estimate (95% CI) | *P* value | *P*_int_ |
| *LA and LV geometry* |  |  |  |  |  |  |  |
| LA volume index, +7.8 ml/m² | 118.1 (54.8 to 181.4) | 0.0003 | 67.8 (-9.02 to 144.7) | 0.083 | 212.1 (104.4 to 319.7) | 0.0001 | 0.0065 |
| LV mass index, +20 g/m² | -55.9 (-120.3 to 0.84) | 0.088 | -95.0 (-168.3 to -21.8) | 0.011 | 28.0 (-93.0 to 148.9) | 0.65 | 0.047 |
| EDV index, +10 ml/m² | 107.6 (45.6 to 169.6) | 0.0007 | 79.2 (4.11 to 154.4) | 0.039 | 158.4 (51.8 to 265.0) | 0.0038 | 0.22 |
| ESV index, +5 ml/m² | 82.5 (18.6 to 146.4) | 0.012 | 34.6 (-41.6 to 110.9) | 0.37 | 157.5 (45.0 to 270.0) | 0.0064 | 0.061 |
| Stroke volume, +15 ml | 109.7 (39.3 to 180.1) | 0.0024 | 95.1 (14.2 to 176.1) | 0.022 | 146.9 (15.8 to 278.0) | 0.028 | 0.84 |
| Ejection fraction, +5.1% | 7.68 (-46.9 to 62.2) | 0.78 | 37.4 (-31.2 to 105.9) | 0.28 | -16.7 (-106.2 to 72.8) | 0.71 | 0.23 |
| *LV diastolic function* |  |  |  |  |  |  |  |
| E peak, +15 cm/s | 155.0 (90.5 to 220.0) | <0.0001 | 107.8 (25.4 to 190.2) | 0.011 | 244.1 (141.9 to 346.2) | <0.0001 | 0.37 |
| A peak, +15 cm/s | 88.3 (10.2 to 166.3) | 0.027 | 83.7 (-20.1 to 187.5) | 0.11 | 89.4 (-30.9 to 209.6) | 0.14 | 0.27 |
| E/A ratio, +0.45 | 107.9 (17.2 to 198.6) | 0.020 | 71.3 (-42.0 to 184.5) | 0.22 | 189.3 (38.7 to 340.0) | 0.014 | 0.55 |
| e’ peak, +3.2 cm/s | 141.6 (48.9 to 234.2) | 0.0029 | 182.3 (68.6 to 296.0) | 0.0018 | 118.7 (-41.5 to 278.9) | 0.15 | 0.063 |
| a’ peak, +2.0 cm/s | 43.3 (-28.5 to 115.1) | 0.24 | 91.3 (6.03 to 176.6) | 0.036 | -42.1 (-165.1 to 80.9) | 0.50 | 0.60 |
| E/e’ ratio, +2.1 | 43.5 (-23.7 to 110.6) | 0.20 | -43.0 (-143.4 to 57.4) | 0.40 | 98.7 (6.76 to 190.6) | 0.036 | 0.0094 |

Parameter estimates (95% confidence interval) indicate changes in ejection work density associated with a 1 standard deviation increase in arterial index. All parameter estimates were adjusted for age, sex, heart rate and body height and weight. Adjustment for BSA-indexed measures did not include body height and weight. P_int_ indicates the P values for the interaction between echocardiographic indexes and sex. BSA indicates body surface area; EDV, end-diastolic volume; ESV, end-systolic volume; LA, left atrial; LV, left ventricular; PWT, posterior wall thickness.

TABLE S5. **Multivariable-Adjusted Associations between Ejection Work Density and Echocardiographic Indexes of Left Ventricular Diastolic and Systolic Function** **in 264 Subjects not Receiving Anti-Hypertensive Treatment.**

|  | Ejection Work Density (Pa) | | | | | | | | |  |
| --- | --- | --- | --- | --- | --- | --- | --- | --- | --- | --- |
|  | All (n=264) | | | Men (n=143) | | | Women (n=121) | | |  |
| *LV diastolic function* | Parameter estimate (95% CI) | *P* value | Parameter estimate (95% CI) | | *P* value | Parameter estimate (95% CI) | | *P* value | *P*_int_ | |
| E peak, +15 cm/s | 60.7 (35.9 to 85.6) | <0.0001 | 47.1 (17.8 to 76.5) | | 0.0019 | 96.9 (57.7 to 136.1) | | <0.0001 | 0.89 | |
| A peak, +15 cm/s | 49.8 (14.7 to 84.9) | 0.0056 | 47.7 (-4.05 to 91.3) | | 0.033 | 40.4 (-16.3 to 97.0) | | 0.16 | 0.083 | |
| E/A ratio, +0.45 | 37.1 (2.68 to 71.6) | 0.035 | 26.7 (-14.1 to 67.4) | | 0.20 | 77.8 (20.8 to 134.7) | | 0.0079 | 0.11 | |
| e’ peak, +3.2 cm/s | 38.2 (2.84 to 73.6) | 0.034 | 54.2 (14.5 to 93.8) | | 0.0078 | 42.8 (-19.9 to 105.6) | | 0.18 | 0.0039 | |
| a’ peak, +2.0 cm/s | 14.3 (-14.5 to 43.1) | 0.33 | 36.0 (3.32 to 68.6) | | 0.031 | -23.9 (-71.6 to 23.9) | | 0.32 | 0.82 | |
| E/e’ ratio, +2.1 | 34.8 (7.08 to 62.5) | 0.014 | 8.68 (-28.9 to 46.3) | | 0.65 | 49.2 (9.71 to 88.7) | | 0.015 | 0.0031 | |

Parameter estimates (95% confidence interval) indicate changes in ejection work density associated with a 1 standard deviation increase in arterial index. All parameter estimates were adjusted for age, sex, heart rate and body height and weight. P_int_ indicates the P values for the interaction between LV diastolic indexes and sex. LV indicates left ventricular.
